# Intestinal helminths infection of rats (*Ratus norvegicus*) in the Belgrade area (Serbia): the effect of sex, age and habitat*

**DOI:** 10.1051/parasite/2011182189

**Published:** 2011-05-15

**Authors:** M. Kataranovski, I. Mirkov, S. Belij, A. Popov, Z. Petrović, Z. Gačić, D. Kataranovski

**Affiliations:** 1 Department of Ecology, Institute for Biological Research “Siniša StankoviĆ”, University of Belgrade Belgrade Serbia; 2 Institute of Meat Hygiene and Technology Belgrade Serbia; 3 Institute for Multidisciplinary Research Belgrade Serbia

**Keywords:** *Rattus norvegicus*, intestinal helminths, nematodes, cestodes, Serbia, *Rattus norvegicus*, helminthose intestinale, nématode, cestode, Serbie

## Abstract

Gastrointestinal helminths of Norway rat (*Rattus norvegicus*) from the Belgrade area were studied as a part of a wider ecological research of rats in Serbia (data on the distribution, population ecology, economic and epizoothiological-epidemiological importance, and density control). Rats were captured from May 2005 to July 2009 at both urban and suburban-rural sites. Of a total of 302 trapped rats 48% were males and 52% females, with 36.5% and 38.8% of juvenile-subadult individuals, per sex respectively. Intestinal helminth infection was noted in 68.5% of rats, with a higher prevalence in male hosts and in adult individuals. Higher numbers of infected juveniles-subadults were noted in suburban-rural habitats, while an opposite tendency was noted in adult rats. Seven helminth species were recovered, of which five were nematode (*Heterakis spumosa*, *Nippostrongylus brasiliensis*, *Capillaria* sp., *Trichuris muris* and *Syphacia muris*) and two cestode species (*Hymenolepis diminuta* and *Rodentolepis fraterna*). The most prevalent parasites were *Heterakis spumosa* (36.7%) and *Hymenolepis diminuta* (30.5%). Sex and habitat-related differences were noted in the prevalence of infection with *Capillaria* sp. and *Trichuris muris*, while there were no age-related differences in the prevalence of infection with any individual helminth species. Significantly higher prevalence of infection was noted in summer as compared to spring or winter, with a tendency to be higher in autumn as compared to spring. The only significant difference in the prevalence of infection between habitat-related was noted during spring. *H. spumosa* was most prevalent in summer, while *H. diminuta* and *N. brasiliensis* in autumn. The mean intensity of infection with *H. spumosa*, *R. fraterna*, *S. muris* and *T. muris* was higher in autumn than in the other seasons, while *N. brasiliensis* and *Capillaria* sp. occured in winter. No more than four helminth species were found in one host.

## Abstract (fr)

Les helminthes digestives de *Rattus norvegicus* de la région de Belgrade ont été étudiées dans le cadre d’une vaste recherche menée sur les rats en Serbie (répartition et écologie des populations, conséquences économiques et épizoo-épidémiologiques, contrôle des densités de populations). Les rats ont été capturés entre mai 2005 et juillet 2009 en zones urbaines et périurbaines et rurales. Sur un total de 302 captures, on comptait 48 % de mâles et 52 % de femelles, avec respectivement 36,5 % et 38,8 % d’individus juvéniles ou préadultes. Au moins une helminthose intestinale a été retrouvée chez 68,5 % des rats, avec une fréquence plus élevée chez les mâles et les adultes. Un nombre plus important de juvéniles et de préadultes infectés a été observé en zones suburbaines et rurales, avec une tendance inverse chez les adultes. Sept espèces d’helminthes ont été retrouvées, dont cinq nématodes (*Heterakis spumosa*, *Nippostrongylus brasiliensis*, *Capillaria* sp., *Trichuris muris* et *Syphacia muris*) et deux cestodes (*Hymenolepis diminuta* et *Rodentolepis fraterna*), les plus fréquents étant *Heterakis spumosa* (36,7 %) et *Hymenolepis diminuta* (30,5 %). Des variations liées au sexe et à l’habitat ont été notées pour *Capillaria* sp. et *Trichuris muris*, mais sans différence liée à l’âge pour chacune des helminthoses. La fréquence des infections était plus importante en été qu’au printemps et en hiver, avec une tendance à être plus élevée en automne qu’au printemps. La seule différence significative selon l’habitat a été observée au printemps. *H. spumosa* a été plus fréquemment retrouvé en été, *H. diminuta* et *N. brasiliensis* en automne. L’intensité moyenne des infections était plus importante en automne pour *H. spumosa*, *R. fraterna*, *S. muris* et *T. muris*, et en hiver pour *N. brasiliensis* et *Capillaria* sp. Un maximum de quatre helminthes a été observé chez un même individu.

Norway rat, *Rattus norvegicus* (Berk, 1769), is a cosmopolitan rodent species with a wide distribution in urban and suburban-rural habitats, commonly found living near sources of food and water, such as refuse and drainage ditches, streams or sewers (Kataranovski, 1999). Because of its high ability to harbor many zoonotic agents, wild Norway rats play a significant role as definitive and/or intermediate hosts for vector-borne animal and human diseases (Bradshaw, 1999; Battersby *et al.*, 2002; Easterbrook *et al.*, 2007). The diversity of distribution of Norway rats in urban, suburban and particularly rural areas, and consumption of a variety of foods as well as materials of human and animal origin, attribute to their exposure to diverse parasitic infections.

While reports of gastrointestinal helminthic parasites of populations of *R. norvegicus* from temperate regions of Europe are numerous (Feliu *et al.*, 1985, 1997; Webster & Macdonald, 1995; Webster, 1997; Ceruti *et al.*, 2001; Stojčević et al., 2004; Redrobe & Patterson-Kane, 2005), there is a lack of data concerning intestinal helminth fauna in *R. norvegicus* in Serbia. Wild *R. norvegicus* is the dominant rat species in this area (Kataranovski, 1999), and represents an important pest rodent. Recently, data on *Calodium hepaticum* (= *Capillaria hepatica*) and *Taenia* (= *Hydatigera*) *taeniaeformis* larvae (*Cysticercus fasciolaris*) liver infections in *R. norvegicu*s were reported (Kataranovski *et al.*, 2010), which represent the first record of these parasites in wild Norway rats in Serbia.

The aim of this study was to examine intestinal helminth fauna of Norway rats from Belgrade area and to investigate the impact of internal (host) factors such as sex and age as well as external factors including different environments (urban or suburban-rural) and season on the prevalence of helminth infection.

## Materials and Methods

A total of 302 rats were captured during four consecutive years, from May 2005 to July 2009. The sample includes all the seasons in the studied years. Rats were collected from different sites (urban and suburban-rural) of the Belgrade area (44° N, 20° E, approximate geometric center of Belgrade 44°49’14” N, 20°27’44” E). Urban sites of Belgrade area were characterized by high population density. Suburban-rural sites of the Belgrade area are with lower population density situated at the outskirts of urban site or small isolated areas of open country with sporadic houses and crofts (barns, stables, pigsties, chicken coops, pens). Animals were captured using snap live traps (14 × 16.5 × 32 cm). Live traps were baited with pieces of smoked bacon and/or fresh-water fish, and were active for five consecutive days per three urban and three suburban-rural stations and season. The captured rats were transported to the animal facility of the Institute for Biological Research “Siniša StankoviĆ”, Belgrade and examined 24-48 hours after trapping. Animal procedures were carried out in adherence to the Ethical Committee of the Institute for Biological Research “Siniša StankoviĆ”, Belgrade. The animals were fed commercial rodent feed and had access to water *ad libitum*. After 24-48 hours the rats were euthanized by barbiturate anesthesia overdose.

For each rat examined, the data on trapping locality, body length (head and body), weight and sex were noted. Rats were classified into juveniles-subadults (< 2.5 months old) and adults (> 2.5 months old) according to body weight (borderline value 200 g) and the weight of the dry eye lens pairs (14.3 mg) as shown previously (Kataranovski *et al.*, 1994).

The material was analyzed using standard parasitological procedures. The stomach, small intestine, cecum and colon were separated from the surrounding fat tissue and placed into individual Petri dishes containing saline. They were opened longitudinally and examined for helminth parasites. Parasites were carefully removed, identified and counted under a stereoscopic microscope (Kruss and Olympus BO61 binoculars and Olympus CHC and Carl Zeiss). The identification of helminths of Norway rat was based on Key To Helminths of Rodents of the Fauna of the USSR (1978, 1979) and descriptions given by Genov (1984). The parasitological terminology and quantitative parameters were according to Buch et al. (1997). Quantitative descriptors of parasite infection were calculated, including prevalence or percent infected, percent infestation, extent of infection, extensity (P = n/Z × 100), mean intensity of infection (MI = N/n) and mean abundance of infection (MA = N/Z) where: n = number of animals (hosts) infected, N = total number of parasites, and Z = total number of animals infected and non-infected. Also, according to Kisielewska (1970), the infection index or invasion index (I = N × n/Z2) was calculated. Statistical analysis was performed using difference between two proportions and Mann-Whitney U-test (STATISTICA 6.0, StatSoft Inc., Tulsa, Oklahoma, USA).

The studied material has been stored in the collection of the Helminthologic section, Department of Ecology, Institute for Biological Research, “Siniša StankoviĆ”, Belgrade, Serbia.

## Results

Of all Norway rats, 48.0% were males and 52% were females. All rats were separated in two age groups, juvenile-subadult (37.4%) and adult (62.6%). Intestinal helminths were found in 207 rats (68.5%). A higher prevalence of infection was noted in male compared to female rats, owing to infection in animals of this sex from both localities (Table I). Higher prevalence of infection in males is mainly due to juvenile-subadults and adults in urban and suburban-rural habitats, respectively.

**Table I.** Prevalence of intestinal helminth infection in rats of different sex, age and from different habitats.

	Males	Females	p
Number of rats	145	157	
Infected	76.5 *	61.1	
Urban	56.6	47.8	
Infected	81.7 *	68.0	0.0490
Suburban-rural	43.4	52.2	
Infected	69.8 *	54.9	0.0339

Juvenile-subadult rats	36.5	38.2	
Infected	67.9	55.0	0.0800
Urban	46.9	51.7	
Infected	81.6 *	61.3	0.0324
Suburban-rural	23.8	48.3	
Infected	33.3	48.3	0.1732

Adult rats	63.4	61.8	
Infected	81.5 * #	64.9	0.0055, 0.0324
Urban	47.8	45.4	
Infected	78.3	72.7 #	0.2750, 0.0342
Suburban-rural	90.6	88.3	
Infected	81.2 *	58.5	0.0076

Significantly different from females at

* p < 0.05, from juvenilesubadults at

# p < 0.05 (3rd column).

Helminthological analysis showed the presence of seven species of parasites, as follows: five Nematoda species – *Heterakis spumosa* Schneider 1866, *Nippostrongylus brasiliensis* (Travassos, 1914), *Capillaria* sp. (Zeder, 1800), *Syphacia muris* (Yamaguti, 1935) and *Trichuris muris* (Schrank, 1788) – and two Cestoda species – *Hymenolepis diminuta* (Rudolphi, 1819) and *Rodentolepis fraterna* (Stilles, 1906). Data on the prevalence, index of infection, mean infection intensity and mean abundance of gastrointestinal nematodes and cestodes in male and female *R. norvegicus* hosts from urban and suburban-rural habitats separately, are presented in Table II.

**Table II.** Quantitative indices of individual intestinal helminth infections of *Rattus norvegicus*.

	Helminth species		n	N	Z	P %	I	MI	MA
(Part 1)	*Heterakis spumosa*	male	54	437	145	37.2	1.1224	8.1	3.0
		female	57	460	157	36.3	1.0637	8.1	2.9
	*Hymenolepis diminuta*	male	50	319	145	34.5	0.7586	6.4	2.1
		female	42	261	157	26.7	0.4447	6.2	1.7
	*Rodentolepis fraterna*	male	22	132	145	15.2	0.1381	9.0	0.9
		female	16	87	157	10.2	0.0565	5.4	0.5

Combined habitats	*Nippostrongylus brasiliensis*	male	27	370	145	18.6	0.4751	13.7	2.5
		female	22	307	157	14.0	0.3212	13.9	2.0
	*Capillaria sp.*	male	14	126	145	9.7 *	0.0839	9.0	0.9
		female	4	55	157	2.5	0.0089	13.7	0.3
	*Trichuris muris*	male	11	49	145	7.6	0.0256	4.4	0.3
		female	7	37	157	4.5	0.0105	8.3	0.2
	*Syphacia muris*	male	6	50	145	4.1	0.0142	8.3	0.3
		female	7	47	157	4.5	0.0133	6.7	0.3

(Part 2)	*Heterakis spumosa*	male	29	242	81	35.8	1.0696	8.3	3.0
		female	26	220	76	34.2	0.9903	8.5	2.8
	*Hymenolepis diminuta*	male	34	187	81	42.0	0.9691	5.5	2.3
		female	23	117	76	30.3	0.4659	5.1	1.5
	*Rodentolepis fraterna*	male	16	73	81	19.7	0.1780	4.6	0.9
		female	12	69	76	15.8	0.1434	5.7	0.9

Urban habitat	*Nippostrongylus brasiliensis*	male	13	146	81	16.0	0.2893	11.2	1.8
		female	10	127	76	13.2	0.2199	12.7	1.7
	*Capillaria sp.*	male	12	127	81	14.8 * #	0.2323	10.6	1.6
		female	3	27	76	3.9	0.0140	9.0	0.4
	*Trichuris muris*	male	7	36	81	8.6	0.0384	5.1	0.4
		female	6	28	76	7.9 #	0.0291	4.7	0.4
	*Syphacia muris*	male	6	53	81	7.4	0.0485	8.8	0.6
		female	5	38	76	6.6	0.0328	7.6	0.5
	*Heterakis spumosa*	male	25	217	64	39.1	1.3245	8.7	3.4
		female	31	218	81	38.3	1.0300	7.0	2.7
	*Hymenolepis diminuta*	male	16	129	64	25.0	0.5039	8.1	2.0
		female	19	147	81	23.5	0.4257	7.7	1.8
	*Rodentolepis fraterna*	male	6	44	64	9.4	0.0645	7.3	0.7
		female	4	33	81	4.9	0.0201	8.2	0.4

Suburban-rural habitat	*Nippostrongylus brasiliensis*	male	14	209	64	21.9	0.7144	14.9	3.3
		female	12	195	81	14.8	0.3567	16.2	2.4
	*Capillaria sp.*	male	2	18	64	3.1	0.0088	9.0	0.3
		female	1	7	81	1.2	0.0011	7.0	0.1
	*Trichuris muris*	male	4	18	64	6.2	0.0176	4.5	0.3
		female	1	4	81	1.2	0.0006	4.0	0.1
	*Syphacia muris*	male	0	0	64	0.0	0.0000	0.0	0.0
		female	2	6	81	2.5	0.0018	3.0	0.1

1 Part 1: significantly different from females at * p < 0.05;

2 Part 2: significantly different from females at * p < 0.05;from urban habitats at # p < 0.05.

The most prevalent were nematodes *H. spumosa* (36.7%) but with relatively lower occurrence (MI = 8.1, MA = 3.0) and *N. brasiliensis* (16.2%) with much higher MI (13.8) but lower MA (2.2), and cestodes *H. diminuta* (30.5%) with lower MI and MA (6.3 and 1.9, respectively) and *R. fraterna* (12.6%) with approximately the same MI as the previous one, but with a much smaller MA (0.7). The species *Capillaria* sp., *T. muris* and *S. muris* had a prevalence below 6%, but relatively high MI (10.1, 4.8 and 7.5, respectively) and lower MA (0.6, 0.3 and 0.3, respectively). No host age or sex-associated differences in the prevalence of infection were found for individual helminth species, except for infections with *Capillaria* sp. The prevalence of *Capillaria* sp. was higher in males than in females, mainly due to infected males caught at urban localities. In contrast, the noted tendency (p = 0.06) of a higher prevalence of *T. muris* in urban *versus* suburban-rural habitats was due to infected females.

According to the infection (invasion) index, the dominant species of helminths in the sample were *H. spumosa* (1.09), *H. diminuta* (0.59) and *N. brasiliensis* (0.36). The influent species were *R. fraterna* (0.09), *Capillaria* sp. (0.04), *T. muris* (0.02) and *S. muris* (0.01).

When prevalence of helminthic infection during different seasons was analyzed the following data were obtained: spring (59.8%), summer (81.1%), autumn (73.3%) and winter (66.0%). Significantly higher prevalence of infection was noted in summer as compared to spring (p = 0.014) or winter (p = 0.019), with a tendency to be higher in autumn as compared to spring. No statistically significant differences were noted between prevalence of infection in rats captured in urban habitats in spring (70.9%), summer (81.2%), autumn (68.7%) and winter (77.3%), while significantly higher prevalence of helminthic infection was noted in summer (80.0%) as compared to spring (43.2%; p = 0.045), in autumn (78.6%) as compared to spring (p = 0.027) as well as in winter (63.1%) as compared to spring (p = 0.042) in suburban-rural habitats. The only significant difference in the prevalence of infection between habitat-related was noted during spring (p = 0.009). Seasonal changes in the prevalence, index of infection, mean infection intensity and mean abundance of helminths in *R. norvegicus* from urban and suburban habitats are presented in Table III.

**Table III.** Seasonal quantitative indices of individual intestinal helminth infection of *Rattus norvegicus*.

		Spring				Summer				Autumn				Winter			
Helminth species		P %	I	MI	MA	P %	I	MI	MA	P %	I	MI	MA	P %	I	MI	MA
*Heterakis*	U	30.9	0.7811	8.2	2.5	41.5	1.4570	8.44	3.5	20.0	0.3733	9.3	1.7	36.4	1.1074	8.4	3.1
*spumosa*	R	24.3	0.4668	7.9	1.9	23.1	7.7692	7.7	7.8	27.3	0.6445	8.7	2.4	36.9	1.0412	7.6	2.8

*Hymenolepis*	U	41.8 #	0.9200	5.3	2.2	29.2	0.4587	5.37	1.6	40.0	0.8267	5.2	2.1	40.9	0.9298	5.6	2.3
*diminuta*	R	21.6	0.3623	7.7	1.7	23.1	0.4083	7.7	1.8	36.4	1.0579	8.0	2.9	23.8	0.4507	7.9	1.9

*Rodentolepis*	U	10.9 #	0.0615	5.2	0.6	23.1	0.2663	5.0	1.2	13.3	0.0978	5.5	0.7	22.7	0.2583	5.0	1.1
*fraterna*	R	2.7	0.0051	7.0	0.2	0.0	0.0	0.0	0.0	0.0	0.0	0.0	0.0	10.7	0.0893	7.8	0.8

*Nippostrongylus*	U	9.1	0.0975	11.8	1.1	16.9	0.3411	11.9	2.0	33.3 *	3.7333	11.2	3.7	9.1	0.1116	13.5	1.2
*brasiliensis*	R	8.1	0.1030	15.7	1.3	23.1	0.8343	15.7	3.6	45.5 *	3.1818	15.4	7.0	17.9	0.4953	15.5	2.8

*Capillaria* sp.	U	9.1	0.0830	10.0	0.9	13.8	0.1981	10.3	1.4	0.0	0.0	0.0	0.0	4.5	0.0227	11.0	0.5
	R	0.0	0.0	0.0	0.0	0.0	0.0	0.0	0.0	18.2 * °	0.3140	9.5	1.7	1.2	0.0125	8.0	0.1

*Trichuris*	U	9.1	0.0397	4.8	0.4	7.7	0.0296	5.0	0.4	20.0 °	0.2000	5.0	1.0	0.0	0.0	0.0	0.0
*muris*	R	2.7	0.0029	4.0	0.1	7.7	0.0296	5.0	0.4	9.1	0.0413	5.0	0.4	2.4	0.0023	4.0	0.1

*Syphacia*	U	5.5	0.0248	8.3	0.4	4.6	0.0178	8.3	0.4	20.0 **	0.3200	8.0	1.6	9.1 #	0.0702	8.5	0.8
*muris*	R	0.0	0.0	0.0	0.0	7.7	0.0115	2.0	0.2	9.1	0.0331	4.0	0.4	0.0	0.0	0.0	0.0

U: urban; R: suburban-rural; significantly different (p < 0.05) from:

* spring,

** summer,

° winter,

# suburban-rural.

When seasonal-related changes in the prevalence of the dominant helminth species was analyzed, *H. spumosa* was most prevalent in summer, while *H. diminuta* and *N. brasiliensis* in autumn. The following prevalence of these helminths was noted during different seasons in urban and suburban-rural habitats, respectively: in spring – *H. diminuta* (41.8% and 21.6%), *H. spumosa* (30.9% and 24.3%) –, in summer – *H. diminuta* (41.5% and 23.1%), *H. spumosa* (41.5% and 23.1%) and *N. brasiliensis* (0.0% and 23.1%) –, in autumn – *H. diminuta* (40.0% and 36.4%) and *N. brasiliensis* (33.3% and 45.5%) –, in winter – *H. diminuta* (40.9% and 23.8%) and *H. spumosa* (36.4% and 36.9%). The prevalence of *N. brasiliensis* was significantly higher in autumn as compared to spring (p = 0.0197 and p = 0.005) in rats from urban and suburban-rural habitats, respectively. The prevalence of *Capillaria* sp. was higher in autumn as compared to spring (p = 0.011) and in autumn compared to winter (p = 0.003) in rats from suburban-rural habitats. No infection with *Capillaria* sp. was noted during autumn in rats captured in urban habitats and in rats captured in suburban-rural habitats during spring and summer. Higher prevalence (p = 0.035) of *T. muris* in autumn compared to winter was noted in rats from urban habitats. This species was not detected in winter. Higher prevalence of *S. muris* was noted in autumn compared to summer in urban rats. This species was not detected in rats from suburban-rural habitats in spring and summer. *R. fraterna* was not detected in spring in individuals from suburban-rural habitats. Significantly higher prevalence (p = 0.047 and 0.049) in urban compared to suburban-rural habitats were noted for *H. diminuta* and *R. fraterna* in the spring and for *S. muris* (p = 0.006) in the winter. The mean intensity of infection with *H. spumosa*, *R. fraterna*, *S. muris* and *T. muris* was higher in autumn than in other seasons, while the higher mean intensity of infection with *N. brasiliensis* and *Capillaria* sp. was noted in winter.

No more than four parasite species were found in one host. Parasitism involving only one species was found in 51.7% of the infected rats. Two species of parasites were found in 31.9% of the infected rats, three species in 14.0%, and four species in 2.4%.

## Discussion

In this study, intestinal helminthic infection of Norway rats from Belgrade area was explored in the context of host sex, age as well as different habitats (urban and suburban-rural) and season. In concordance with the data showing that wild small rodents rarely remain uninfected (Behnke *et al.*, 2001), our study showed a high prevalence of infection with intestinal helminths in wild Norway rats. It might be ascribed to high reproductive potential/ high population density, relatively small home range and radius of activity, and omnivorous way of nutrition (Hrgović *et al.*, 1991). In addition, their neighborhood to domestic animals might contribute. Higher prevalence and intensity of intestinal helminthic infection of male compared to female rats and many other small rodents (Ims, 1987; Poulin, 1996; Shalk & Forbes, 1997; Moore & Wilson, 2002; Kataranovski *et al.*, 2008) may be attributed to the fact that infected males have larger territories than uninfected males (Brown *et al.*, 1994a) and that the home range of males tend to overlap, which could increase their exposure to infection, while reproductive females show a stronger site-specific organization which could explain low rates of transmission (Davis *et al.*, 1948; Pisano & Storer, 1948; Calhoun, 1962; Ims, 1987). In addition, the negative impact of the male hormone testosterone on immune defense functions (Grossman, 1989; Folstad & Karter, 1992) may account for a greater propensity of males for helminth infection. Higher prevalence of infection among male rats may be explained by the hypothesis that, among mammals, the larger bodies of males are easier targets for parasites (Arneberg, 2002). Brown *et al.* (1994a) proved an overdispersed distribution of *Heligmosomoides polygyrus* with higher prevalence of infestation in males and heavier individuals of *Apodemus sylvaticus*. The infected rodents moved more often and faster than uninfected rodents (Brown *et al.*, 1994b). The reasons for these results are still unclear (Klimpel *et al.*, 2007). Age-related differences in the prevalence of infection may reflect the fact that older rats have a longer exposure time to potential infection (Easterbrook *et al.*, 2007). The underlying mechanism(s) of the higher prevalence of *Capillaria* sp. and *T. muris* in urban habitats is not known at present, but warrants future attention.

The results of this study showed that *R. norvegicus* from Belgrade area is host to five nematode and two cestode species. This is in line with data which showed that wild Norway rats harbour several helminth species (Webster & Macdonald, 1995; Battersby *et al.*, 2002; Gomez Villafañe *et al.*, 2008). The results of our study are the first records of intestinal helminth fauna of wild *R. norvegicus* in Serbia, along with recently noted *C. hepaticum* and *T. taeniaeformis* liver infections in *R. norvegicus* (Kataranovski *et al.*, 2010).

The monoxenous nature of the life cycle of nematodes may be responsible for this parasitic group dominating the helminth community of wild rats, as parasites with simple and direct life cycles may have more chance to follow the dispersion of their hosts than parasites with indirect life cycles (Bellocq *et al.*, 2003). The longevity of *H. diminuta* in its normal mammalian host may contribute to the high prevalence of infection of rats, as once established, it can live as long as its host (Read, 1967). No trematode species was found in the intestines of wild Norway rats, in line with the data from the neighboring country of Croatia (Stojčević *et al.*, 2004). Indeed, these helminths are rare in other geographic areas as well, according to the data from Asia (Seo *et al.*, 1968; Seong *et al.*, 1995; Paramasvaran *et al.*, 2009).

Moderate prevalence of *H. spumosa* (36.7%) is in accordance with the results of Seo *et al.* (1968) in South Korea and Stojčević *et al.*, 2004 in Croatia. Research results of Firlotte (1948) in Canada and Tscherner (1996) in Germany, as well as in Argentina (Gomez Villafañe *et al.*, 2008) showed, however, a high prevalence of *H. spumosa* in *R. norvegicus*. Moderate to high prevalence of *H. diminuta* has been reported in different parts of the world including Kuwait (Zakaria & Zaghloul, 1982), Great Britain (Webster & Macdonald, 1995), Qatar (Abu Madi *et al.*, 2001;^ 2005^), Croatia (Stojčević *et al.*, 2004), Argentina (Gomez Villafañe *et al.*, 2008) and Kuala Lumpur, Malesia, Southeastern Asia (Paramasvaran *et al.*, 2009). Results on *N. brasiliensis* are in accordance with results from other studies (Stojčević *et al.*, 2004; Gomez Vallafane *et al.*, 2008; Paramasvaran *et al.*, 2009). *T. muris* was recorded in our country by Habijan-Mikeš (1990) in *Apodemus flavicollis*, by Kataranovski *et al.* (2008) in *Mus musculus* and by Bjeliććabrilo *et al.* (2009) in *Clethrionomys glareolus*. The low prevalence of this parasite species was attributed by Lewis (1987) to the existence of a strong immune response against this species by hosts, leading to low values of prevalence of the given parasite species in “wild” hosts.

In addition to the significance for parasitological studies in natural ecosystems, our data are of epidemiological importance since some of the detected helminths may occasionally infect humans. In this regard, cases of human infections with *Hymenolepis diminuta* (Sun, 1988; Lalošević *et al.*, 1996; Tena *et al.*, 1998; Marangi *et al.*, 2003; Mowalvi *et al.*, 2008; Watwe & Kaur Dardi, 2008) and *Capillaria* sp. (Lalošević *et al.*, 2008) were reported.

In conclusion, our study contributes to the growing wealth of information on the range and variation in the component community structures of intestinal parasites in wild Norway rats from different regions of the world and from different climatic zones. The important position occupied by these animals in biocenoses, their distribution, population density, the fact that this species cohabitates with humans, and the insignificant knowledge of their gastrointestinal parasites at the territory of Serbia, indicate the necessity of further investigations.
